# Detecting Anomalous Transactions via an IoT Based Application: A Machine Learning Approach for Horse Racing Betting †

**DOI:** 10.3390/s21062039

**Published:** 2021-03-13

**Authors:** Moohong Min, Jemin Justin Lee, Hyunbeom Park, Kyungho Lee

**Affiliations:** 1 Department of Information Security, School of Cybersecurity, Korea University, Seoul 02841, Korea; monglab@korea.ac.kr; 2 Center for Information Security Technology (CIST), Korea University, Seoul 02841, Korea; jeminjustinlee@korea.ac.kr; 3 A3security, Seoul 07217, Korea; hyunbeom.park@a3sc.co.kr

**Keywords:** anomaly detection, big data, cyber security, transaction data, time series data, horse racing, mobile sensors, machine learning, Internet of Things

## Abstract

During the past decade, the technological advancement have allowed the gambling industry worldwide to deploy various platforms such as the web and mobile applications. Government agencies and local authorities have placed strict regulations regarding the location and amount allowed for gambling. These efforts are made to prevent gambling addictions and monitor fraudulent activities. The revenue earned from gambling provides a considerable amount of tax revenue. The inception of internet gambling have allowed professional gamblers to par take in unlawful acts. However, the lack of studies on the technical inspections and systems to prohibit unlawful internet gambling has caused incidents such as the Walkerhill Hotel incident in 2016, where fraudsters placed bets abnormally by modifying an Internet of Things (IoT)-based application called “MyCard”. This paper investigates the logic used by smartphone IoT applications to validate the location of users and then confirm continuous threats. Hence, our research analyzed transactions made on applications that operated using location authentication through IoT devices. Drawing on gambling transaction data from the Korea Racing Authority, this research used time series machine learning algorithms to identify anomalous activities and transactions. In our research, we propose a method to detect and prevent these anomalies by conducting a comparative analysis of the results of existing anomaly detection techniques and novel techniques.

## 1. Introduction

With the advent of digital technologies, the consumption of sports entertainment has drastically increased in recent years. Currently, the global gambling industry is estimated at around 227 billion USD [1], and the online illegal gambling market has increased proportionately to other entertainment industries. Illegal gambling is defined as all forms of gambling not authorized by a given user’s country and consists of illegal sports gambling, black market slot machines, and illegal online gambling [2]. Gambling industry profits are often used to run various leisure services such as ski and golf resorts and contribute to the national revenue and related industry sectors.

Horse racing has changed significantly throughout the history, introducing systems such as pari-mutuel betting [3] and modern calculation methods based on IT systems [4]. In pari-mutuel betting, all invested money is placed in a pool that is distributed to winner(s) after administration costs and taxes have been deducted. Previously, pari-mutuel betting required an advanced calculation system called a totalizator to present the changing odds over time as new bets were submitted; Mobile Horse Racing Betting (MHRB), however, relies instead on Internet of Things (IoT) sensors, devices, and applications. As smartphone applications are more accessible to manipulation by hackers [5], there is a new imperative to develop anomaly detection capabilities to monitor transaction data.

The horse racing industry has become an IoT-based sport, encouraging gambling authorities to establish new software systems using IoT safeguards. In South Korea, a public entity called the Korea Racing Authority (KRA) manages the racing industry and enforces several strict laws. For example, public broadcasts normally cannot show images or videos of horse racing or the KRA logo in order to minimize the public recognition of horse racing. Gambling is only allowed through a IoT based Application called “MyCard”, which validates registered users’ locations using global positioning systems (GPS), Bluetooth, and Wi-Fi sensors to ensure that bets are placed from inside one of the country’s three horse racing stadiums or 30 branches. As of 2021, these bets are limited to 100,000 KRW (approximately 90 USD) per race for individuals.

These limitations have highlighted the risk of fraudulent betting. As betting limits can only be applied to South Korean nationals, foreign citizens have found opportunities to manipulate odds and evade taxes on MyCard. At the Grand Walkerhill Hotel in 2016 and 2017 [5], fraudsters cheated by splitting their bets over many accounts to avoid taxes, which increased depending on the amount of profit. For instance, a customer who purchases 1000 bets of 100 KRW (approximately 0.09 USD) individually would yield a higher return of investment than another customer who would make a single purchase which would equate to 100,000 KRW (approximately 90.99 USD). Under the South Korean tax law, winners must pay 22% of their profit in taxes if the odds rate exceeds 100 times the betting amount, but there are no taxes if the profit is less than 100,000 KRW. Normally, gamblers will purchase bets towards the selling ends, because the probability of guessing the odds at the end of the race has a higher probable chance of winning for the gamblers. This odds ratio is a vital piece of information for gamblers, but fraudsters may hack the MyCard to manipulate the flow of the odds ratio and gain access to unequal information. To prevent these forms of cheating, anomaly detection specifically focused on the purchase transaction process is crucial.

Previous research mainly focused on the minimum and maximum range of sales in gambling to construct a SafeZone [6], but this approach reported too many anomalies. To overcome this limitation, this research pursued further steps to detect and monitor anomalies. Transaction data in horse race gambling shows periodic patterns, so this research focuses on detecting the order of anomalies using machine learning. To summarize, time-series algorithms such as recurrent neural networks (RNNs) and long short-term memory (LSTM) were used to predict bet purchases and suggest a SafeZone that guards against anomalies. These detection methodologies could be applied to many other examples in which data from sensors are obtained in a periodic pattern [7]. If our research were taken further, the theories and principles discussed here could be extended to healthcare with IoT sensors in smartphone devices [8,9,10] and to IoT related sensing systems in the field of power estimation in buildings and land [11].

In Section 2, the background of the research is introduced. In Section 3, previous research and conditions are discussed. In Section 4, the ordering of transactions in mobile applications is analyzed. In Section 5, based on the points made in Section 4, models and ideas for detection are suggested. In Section 6, the experiments are described. In Section 7, implications and future work are discussed.

## 2. Background

### 2.1. Gambling and Horse Racing

The gambling industry has been regarded as an important source of tax revenue by governments in several countries, with the size of the global gambling industry amounting to approximately 227 billion USD [1]. Individuals from Australia spend the highest amount at approximately 1200 USD annually, followed by individuals from Singapore, who spend approximately 1100 USD annually [12]. Often times, the gambling industry is concentrated in a particular region or city, such as Las Vegas and Macao.

There are three major types of gambling: (1) casino gambling, including slot machines, roulette, and baccarat; (2) sports betting in horse racing, soccer, baseball, and basketball; and (3) lottery games, such as scratch-offs and lotto.

Recently, betting on soccer and baseball has become popular. However, a sports betting is generally associated with horse racing. As of November 2019, horse racing made up the largest percentage of the European betting market [13]. There are two categories in horse race betting: fixed odds betting and pari-mutuel betting. A fixed odds betting is organized by bookmakers who design appropriate odds that maintain consumer interest while ensuring their own profit. In contrast, pari-mutuel betting pursues a sustainable running system. It was first suggested by a Spanish entrepreneur, Joseph Oller, in France in the 1860s [14]. In pari-mutuel betting, all invested bets are collected, and all leftover benefits are given to the winners after deduction of the transaction fees, administration costs, and taxes.

Pari-mutuel betting has spread to many countries and regions, as it enables horse racing gambling authorities to secure stable budgets and profits. A key characteristic of pari-mutuel betting is that odds change continuously until betting closes, and that the odds depend on the bets placed. The final odds depend largely on which horse gamblers bet heavily at the end of the sale. A totalizator (tote system) is required for calculating the odds.

### 2.2. Totalizator and Mobile Horse Racing Betting

The first totalizator was invented in Australia in 1913 and has continued to advance over the decades, by increasing its calculation speed [15]. The pari-mutuel formula can be expressed as an equation (1). The bets of each of the gamblers (*Sum_i_*) are summed and the percentage (*p*) of the horse racing company’s take is subtracted. The remaining pool is then divided and paid out by the bets to the winners (*Sum_winner_*). The first totalizator was an automated machine, as shown in Equation (1) [4,16].

(1) $$\frac{\sum Sum_{i}\times \left(1-\frac{p}{100}\right)}{Sum_{winner}}$$

**Figure 1:**
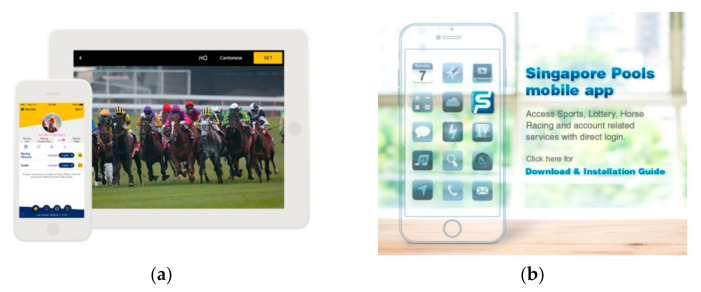
Mobile betting app from (**a**) Hong Kong [19] and (**b**) Singapore [18].

Mechanical totalizators advanced well into the 1960s with enhancements to reliability, new features such as various types of bets, and technical advancements such as memory and logic implementation relays. In the 1970s, computerization of totalizators began [4], and the development of information technologies resulted in significant changes in terms of the devices used for betting. Recently, several countries have legalized mobile horse racing betting (MHRB) or online horse racing betting (OHRB) [17] such as the United Kingdom, Singapore [18], Hong Kong [19], and South Korea, as shown in Figure 1. Likewise, many gambling authorities throughout the world use MHRB applications, which include horse race schedules and outcomes, race records, and the details of individual horse racing players. Currently, transactions through MHRB occupy a bigger share of all betting transactions than traditional methods such as automatic machines or desktop devices.

### 2.3. Horse Racing in South Korea and Internet of Things

In South Korea, two services called “K-TRACK” and “K-TOTE” have been adopted to regulate horse racing. K-TRACK is a real-time tracking system [20] that traces location through ultra-wideband (UWB) communication. The tags distinguish “for racing” and “for training”.

K-TOTE, as a totalizator, provides various types of terminal wagering services, including an IoT-based application. MyCard is a representative IoT based Mobile Horse Racing Betting application. MHRB in South Korea is only allowed on MyCard, which validates each user‘s location, one of the eligibility criteria, by using information provided by GPS, Bluetooth (Beacon), and Wi-Fi sensors [21].

MHRB apps require an account for betting, and the connection location information is recorded for each account, so location validation is essential. Because of the risk of manipulation when using GPS alone, MyCard also uses Bluetooth to validate location.

Bets cannot be placed until location validation is completed. The MyCard application verifies locations including the main race park and branches. To verify a user’s presence in the suburban race park, the system only needs GPS information, but branches require more complex information, as most are located in busier urban areas throughout the country. Wi-Fi scanning and Bluetooth sensors called “iBeacons” offer additional precision to verify a specific indoor location. iBeacon, an IoT device for measuring location indoors proposed by Apple, utilizes Bluetooth Low Energy (BLE) technology [22]. The iBeacon has relatively fewer errors than GPS and can detect distances up to approximately 70 m [23]. Distance was measured most precisely when the range was less than 8 m [24]. The beacons’ interaction with mobile applications enables indoor navigation and proximity navigation [25]. By installing iBeacon on each branch, the MHRB app proves that the installed iBeacon is connected inside the branch.

### 2.4. Threat of MHRB

Location verification allows regulators to observe players’ movements and behaviors inside race parks and branches. Thousands of CCTV units are used to watch for players recording or leaking information such as odds boards. However, if the location can be modified, users can connect accounts to multiple smartphones to avoid taxes or unfairly increase the amount bet, which may affect the real-time dividend rate.

There are many ways to manipulate the MHRB app’s connection location. First, fraudsters can simply abuse the coverage range of the beacon, finding outside points where the app is accessible. Second, GPS information can be obtained and modified directly if the MHRB application is compromised. Finally, beacons can be stolen, copied, controlled, fabricated, or reused [26]. While these weaknesses might suggest that validation logic and hardware need to be strengthened, fraudsters have demonstrated a considerable ability to analyze, reverse engineer, and modify these systems faster than they can be improved [27]. This suggests that a novel view is needed for detecting threats.

### 2.5. Fraud and Anomaly Definitions in MHRB

The term “fraud” refers to abnormal betting meant to evade taxes or avoid rules enforced by accredited gambling agencies. The Walkerhill Hotel incident is taken as a guiding example for this paper due to its abnormal purchase data and government classification as fraud [5]. In the Walkerhill Hotel Seoul incident, MyCard was manipulated to place an abnormal number of bets in order to split the total purchase amount and its corresponding tax liability. An “anomaly” refers to suspicious purchases within a totalizator. This anomaly becomes fraud when a country’s gambling authority confirms the case. These days, totalizators have adapted to the mobile communication environment by using IoT based applications, allowing anomalies to be automatically identified through abnormal patterns in purchase data.

## 3. Previous Research

### 3.1. Fraud Detection Research

Limited research has been conducted on fraud in the horse racing domain, as horse racing has a fixed sale time for betting tickets with limitations on the purchase method and amount. Previous fraud detection research has primarily been conducted on financial entities such as credit card companies. Banks have been widely and professionally investigated with the goal of detecting financial fraud, such as money laundering. Money laundering takes place when illegally obtained money is transferred to another place or group with serious social implications [28]. These crimes are accompanied by complex transactions and financial networks, making detection very difficult [29]. Money laundering also takes place in horse racing, as it is easy to deposit, invest, and withdraw money through gambling.

Prior to the 21st century, credit cards first introduced fraud detection investigations [30]. Several credit card companies have faced financial losses due to fraudulent use of their card products [31]. Recently, financial groups invented a machine learning-based detection technology, which compares naïve Bayes, k-nearest neighbor, and logistic regression [32]. There is an even more advanced approach to handle the ˃75 million card payments within the last three years [33]. There are similarities and differences between the credit card industry and the horse racing industry, with a notable distinction being that credit card payments do not usually occur repeatedly within a very short timeframe. By contrast, horse racing bets can reasonably be made multiple times within a minute, making it impossible to identify fraud by frequency alone and suggesting the need to find other systemic patterns in fraudulent betting. One possible solution to this problem is time-series machine learning. LSTM, a time-series machine learning architecture, has been used to detect fraud in applications ranging from credit card transactions [31] to power plant abnormalities [34]. This study focused on the order of transactions, rather than analyzing each transaction event.

### 3.2. Statistical Fraud Detection in the Horse Racing Gambling

A previous study investigated statistical detection methods in horse racing gambling [6], using data from the Korea Racing Authority to analyze betting patterns. They arranged data at intervals of 15, 30, and 60 min in a graph and then tried to identify a particular set of characteristics or patterns to distinguish normal and abnormal transactions. Specifically, this paper suggested an optimized methodology for periodic transactions based on a comparative analysis of elastic data solutions and anomaly detection. The study defined the flexible window (FW) between the start and end of each race based on transaction flow and suggested a “SafeZone” using a statistical process to distinguish normal and abnormal ranges. Transactions outside this SafeZone were regarded as anomalies or possible fraud. The results showed that the Grand Walkerhill Hotel incident could have been detected, and that a FW could be used for periodic analysis of transaction data in gambling. However, there were limitations; when the SafeZone was set widely, the number of anomalies decreased, and when the SafeZone was set narrowly, the number of anomalies increased even if it was in the normal range. Based on the training period, issues with monitoring arose due to the excessive deviations in the detected number of anomalies. This suggests that the method is not ideal to monitor anomalies simultaneously during a race, so this paper aims to suggest development principles that can better monitor anomalies.

## 4. Analysis of Transactions

### 4.1. Sample Transaction of a Branch of Horse Racing (A Single Race)

**Figure 2:**
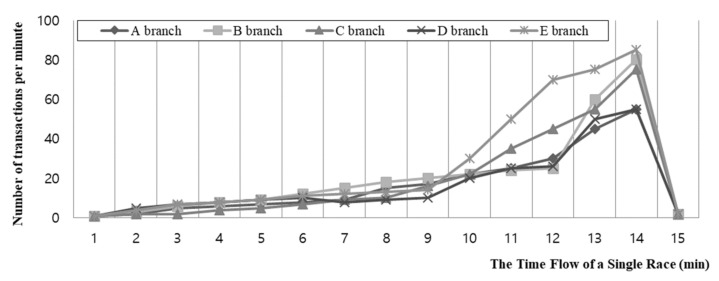
The average bets in a race that occurred in ten branches on the first Friday in January 2020.

This research used betting ticket sales transactions from the Korea Racing Authority. The start and end time of sales in each race can be assumed from the flow of transactions. As shown in Figure 2, sales transactions show that all races are periodic. The sales are initially small, but the number of transactions dramatically increases toward the end. This is a characteristic of pari-mutuel: odds are volatile in the beginning and middle but become similar to payoff odds as the end approaches. The horse racing company ends the betting ticket sales immediately before the start of racing. As can be seen in the graph, the highest number of bets was placed right before the start of the race, after which betting dropped to zero. The numbers of transactions in the ten branches are similar, but the shapes of the graphs are not identical.

### 4.2. Sample Branch Transactions in Horse Racing (One Day of Racing)

**Figure 3:**
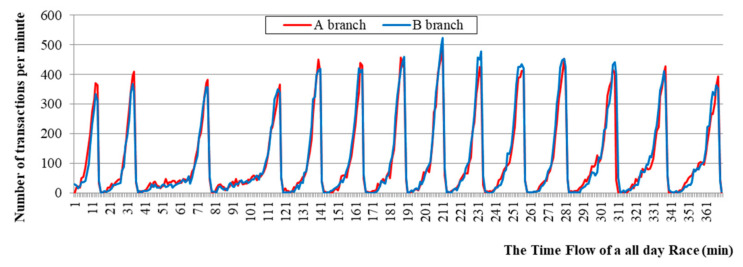
The differences in transactions between each race in two branches.

Figure 3 shows that the shapes of the graphs are similar, but the size of the bets differs depending on the branch. In addition, it is found that the size of the bets increases for the mid races and decreases for the later races. The time gap between races varies from 15 min to 60 min, depending upon scheduling.

### 4.3. RNN, LSTM, and Autoencoder

As the transactions are repeatedly of a similar shape, this paper investigated whether time series machine learning can be utilized. RNN and LSTM are representative time-series machine learning methods for anomaly detection [35]. RNN is a type of neural network that constructs a directed cycle with hidden nodes. RNN is known to show significant loss of learning performance by decreasing the gradient in the backward pass if the distance between the point of information and the point where it uses the information is far. This is called the vanishing gradient problem. LSTM [36] has been suggested as a solution to the vanishing gradient problem in RNN. LSTM adds the cell state in addition to the hidden state of the RNN.

An “autoencoder” encodes particular data through “reconstruction”, which compresses input data and then expands it to produce new data that is similar to the original [37]. This functions as a generative model to produce new data similar to training data.

## 5. Method

### 5.1. Addressing the Issues with a SafeZone

If the SafeZone had been designed using a statistical approach similar to previous research [6], it could have estimated the number of purchases, which is “y”, at the time point of “x”. The normal range of the y value is determined by averaging or the MinMax method. In other words, the threshold is determined by comparing the average of each group or branch, and other data outside of this threshold is regarded as an anomaly.

Previous methods of creating a SafeZone only used this statistical approach. For example, if there were 10–15 races a day on average, each race had a flexible window (FW), and all races on a day overlapped. The FW was aligned at the right side, and therefore peak values did not overlap but were spread on the right side, and this overlapping range was determined to be a SafeZone. Overall, it is necessary to determine the appropriate range and period conditions for an ideal detection principle.

### 5.2. Applied Anomaly Detection Method Using LSTM-AE

It has been suggested that anomaly detection research should adopt an autoencoder (AE) [38,39,40]. The rationale is to compare the time series data to autoencoder output and identify abnormalities if the gap is significant. LSTM-AE is an autoencoder which applied encoder-decoder LSTM architecture to sequence data [41]. This paper applied this methodology to real word data from horse race transactions.

The process of anomaly detection using an autoencoder is:

- Compress input data in a low dimension through encoders.
- Restore the compressed sample to its original dimension through decoders.
- Calculate reconstruction error of input and restored samples.
- The calculated reconstruction error becomes the anomaly score, which is used to identify abnormalities in comparison to a threshold. If the error is larger than the threshold, it is regarded as abnormal. If the error is smaller, it is regarded as normal.

Hence, we can train LSTM autoencoder models using only normal data. The training is performed to minimize the mean squared error (MSE). After this, loss of MSE is calculated on every count, and then the threshold is used to distinguish normal and abnormal data. Based on this threshold, the point of “t + n” in the test set is predicted.

### 5.3. Proposed Anomaly Detection Method Using a SafeZone

This paper suggests a method to optimize transactions in an IoT-based application. Specifically, in this research prediction theories were used in conjunction with typical statistical approaches, and time-series machine learning was used to minimize the number of reported anomalies within the normal range.

#### 5.3.1. Suggestions for SafeLine and SafeZone

The main difference in this approach compared with the traditional statistical approach for fraud detection [6] is how the SafeZone is decided. Herein, the use of time-series algorithms for predicting the size of bets for an improved SafeLine is proposed, which eventually contributes to the development of the SafeZone.

First, the width in the overlap of the data, which is cut by the FW, has been minimized by alignment with the top rather than the right side. Second, data were collected and tested for each race during the training period rather than for a single day because the size of bets in each race was different, as can be seen in Section 4.2. Based on this idea, a prediction line is set through time-series machine learning algorithms, and then a SafeLine is drawn to create the SafeZone. A safe point is then produced each minute. If the prediction value is negative, it is designed to automatically be set to zero in this experiment.

#### 5.3.2. SafeZone Using Fixed Values (Expand SafeLine to SafeZone)

**Figure 4:**
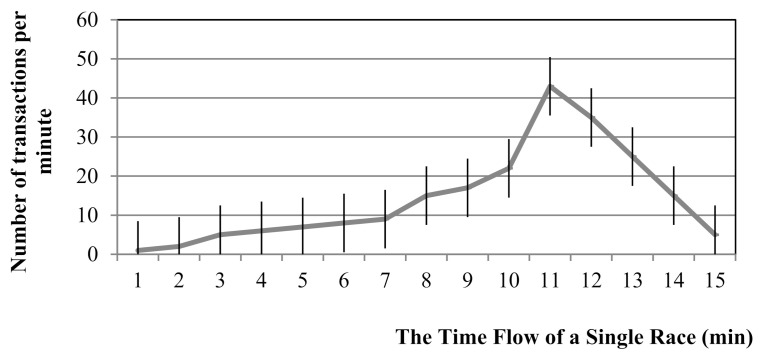
SafeZone using a fixed value.

A SafeZone is created based on the SafeLine information. The upper limit is determined every minute. This can be drawn similar to a stock market chart, as can be seen in Figure 4.

After several trials, it was concluded that 35% of the top value was an ideal fixed value for minimizing anomalies. The fixed value of 35% is an example and could be variable. The fixed values here could be regarded as weight. In Figure 4, the top value of 43 (peak) is found after 11 min. As 35% is a fixed value (plus or minus 15), the criteria range for the SafeLine, which is the base of the SafeZone, is 35% of 43. Then, the upper limit value of the SafeZone is decided. Anomalies are detected if 35% is set as a fixed value, and the peak point is sufficiently high.

#### 5.3.3. Horizontal Movement in the SafeZone

The newly decided SafeZone is arranged by peak value. If several high points that are close to the peak appear, it could be an anomaly. Alternatively, the SafeZone could be moved horizontally to minimize anomalies.

### 5.4. Detecting Anomalies Using the SafeZone

The value of purchased bets is compared to the SafeZone, which is generated by a time-series algorithm, and an anomaly is reported if the value falls outside.

**Figure 5:**
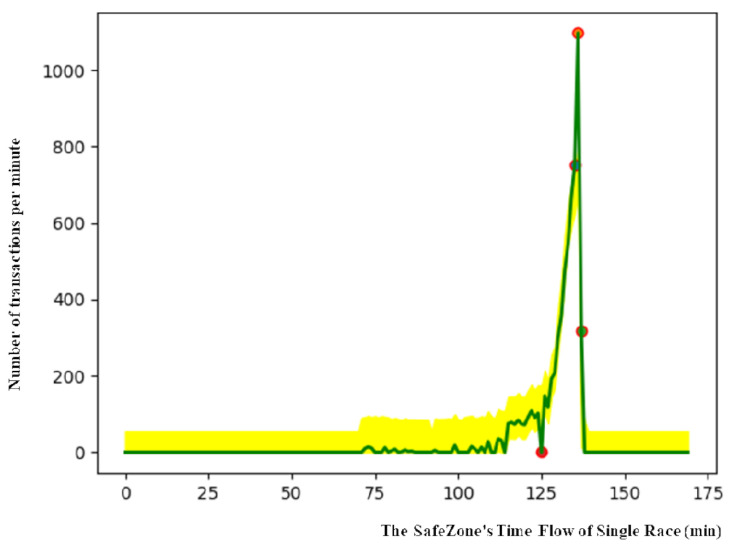
Example of SafeZone and anomaly detection.

The yellow highlighted area represents the SafeZone in Figure 5, and the green line indicates purchased bet values that are added in real time. In Figure 5, the *x*-axis indicates the flow of time, and the *y*-axis represents the number of transactions per minute. As the SafeZone is flexible depending upon the period of training, the time flow that is shown on the *x*-axis does not indicate all time in a single race. In order to ensure that the top value of the SafeZone is aligned with that of a real race, the SafeZone is moved horizontally.

The red circle is a detected anomaly. Monitoring the detection of anomaly points could be a useful solution in real life for protecting games and improving online security in general against anomalies.

**Figure 6:**
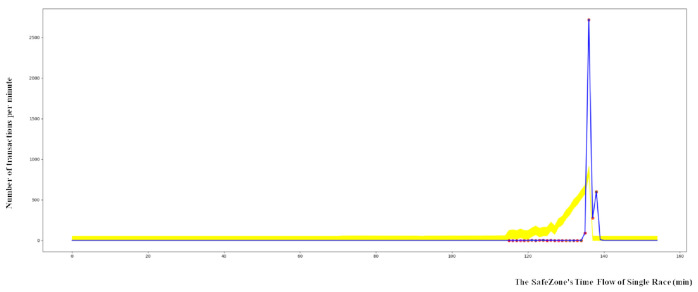
Outcome of a race at Walkerhill with application of the SafeZone.

In the Walkerhill data, a significant number of anomalies were found compared to other branches, indicating systemic fraud. Figure 6 shows outliers in the Walkerhill data.

## 6. Experiments and Evaluation

### 6.1. Preparation Process for Experiments

This evaluation was done in Python using “Keras”, an open-source software library, which creates an interface of artificial neural networks. Other library sources such as sklearn, numpy, pandas, tqdm, matplotlib, pickle were used.

### 6.2. Explanation of Experimental Data

This research performed experiments using Walkerhill data from 3 June 2016 to 22 February 2020. The steps we followed were: Train an LSTM autoencoder on the horse racing transaction data from 13 October 2017 to 22 February 2020, which was a normal period with no anomalies. Then, use the LSTM autoencoder to reconstruct the error of the test data from 3 June 2016 to 1 October 2017. For our evaluation methodology, the cases which exceeded the highest value of the normal period were labeled as fraud.

### 6.3. Comparison of Anomaly Detection Method using LSTM-AE and SafeZone

#### 6.3.1. Experiment on Anomaly Detection Method using LSTM-AE

The experiment with LSTM-AE included the following steps:

#1. The experiment data is counted by minute.
#2. Split train data and test data.
#3. Convert input data into 3D format (samples, timesteps, features). This shape is required for the LSTM network.
#4. We set our network to have a memory of 3 min.
#5. We define the reconstruction LSTM autoencoder architecture. RepeatVector() repeats the inputs 30 times. In addition, set return_sequences = True, so the output will still be a sequence. TimeDistributed(Dense(X_train.shape [2])) is added at the end to get the output, where 2 is the number of features in the input data.

#### 6.3.2. Experiment of Anomaly Detection Method Using SafeZone

**Figure 7:**
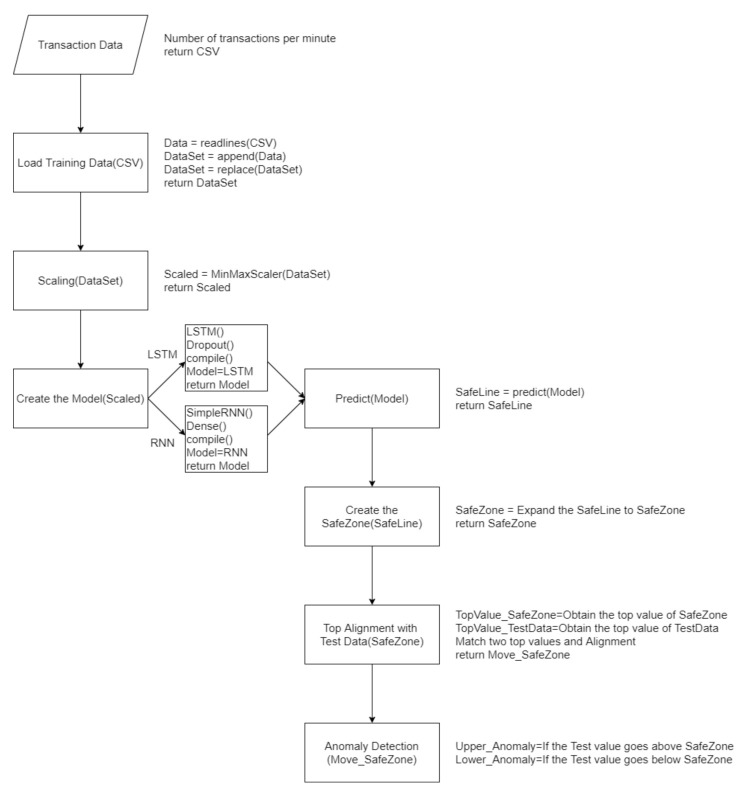
Flow chart of the experimental design using SafeZone

The experiment with SafeZone included the following steps, as shown in Figure 7:

To explain the options that are omitted flow chart in Figure 7, first, an option of a function in “Scaling” sets MinMaxScaler(feature_range = (0, 1)), which means that values are converted into minimum 0 and maximum 1. This was applied to avoid producing negative values. In “Create the Model”, either LSTM can be chosen. The number of cells in both LSTM and RNN are set to 300.

Some detailed settings of the LSTM are as follows:

- regressor.add(dropout (0.5))
- regressor.compile(optimizer = ‘rmsprop’, loss = ‘mean_squared_error’)

Dropout is set to 0.5, which is designed to choose 50% of the input data and let them become value 0 in order to avoid overfitting.

In “compile()”, “RMSprop” was used as the “optimizer” and mean_squared_error was used as the “loss function”. RMSprop is designed to stop the learning rate from dropping, which is a weakness of the “AdaGrad optimizer” and is a common methodology [42,43]. The second parameter, loss function, used mean_squared_error, which judges using the mean value from the square of the real value and predicted value.

One detailed setting for RNN is as follows:

- regressor.add(Dense(units = 150, activation = ‘relu’))

If “dropout” is applied in RNN, it affects memory and may lose important past information, which eventually causes the model’s performance to degrade [44]. Therefore, dropout was not applied in RNN in this research. As for “activation function”, “ReLU” was used to solve the vanishing gradient problem.

#### 6.3.3. Result and Discussion

Table 1 includes the experimental data from Section 6.3.1 and Section 6.3.2 and compares the outcomes of the previous study with the novel methodology in this research. The new methodology with a SafeZone showed better performance than the previous method in several key metrics, including accuracy. Specifically, the SafeZone case (RNN) showed better outcomes than SafeZone (LSTM) in accuracy, precision, recall, and F1 score. Both cases (LSTM and RNN) showed scores as high as twice the original method in precision and recall. These outcomes indicate that the current study’s rationale more accurately detects fraud in the Walkerhill data than the previous method.

This indicates that a SafeZone method using RNN and LSTM can be practically applied to further anomaly detection. It also indicates that a SafeLine made by time-series machine learning algorithms is optimized to build an accurate SafeZone.

### 6.4. Comparison between RNN, LSTM, and Statistical Approaches in SafeZone

This experiment was conducted to change the baseline algorithm that draws the SafeLine and identify the number outside the SafeZone in an ordinary branch without fraud. The purpose of this experiment was to determine the appropriate training period in the monitoring function.

This paper designed the experiment as below.

- Aim: Comparing RNN, LSTM, and statistical approaches
- Training and test data: 10 branches
- Types of training periods:
  - 2016~2019: 4 years
  - 2019: 1 year
  - December 2019: 1 month
  - Last week of December 2019: 1 week
- Test periods”Race 1, Race 2, and Race 3” in the first week of January 2020

**Figure 8:**
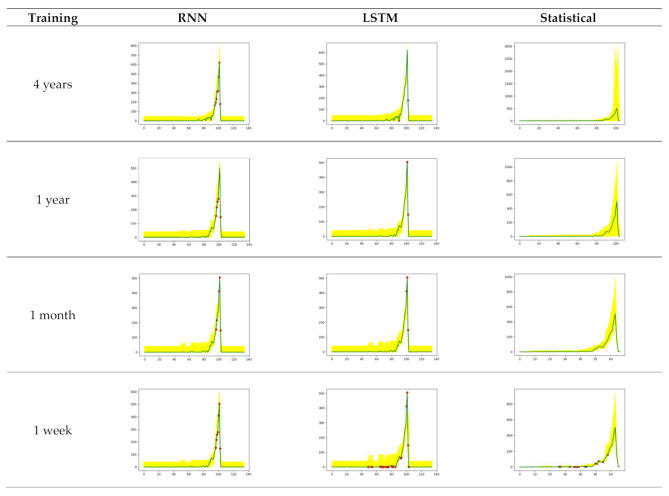
Anomaly detection of sample branch using RNN, LSTM, and statistical approaches.

Four types of training periods were applied to predict the outcomes of three races on Friday in the first week of January 2020. Figure 8 depicts the graphs of a sample branch utilizing RNN, LSTM, and Statistical according to the training data used. In Figure 8, the *x*-axis indicates the flow of time, and the *y*-axis represents the number of transactions per minute.

Table 2 compares the number of anomalies between RNN, LSTM, and statistical approaches. This is the sum of the numbers identified as anomalies per race in 10 branches. When comparing the data in Table 2, it is apparent that the number of anomalies increases as the training period is shortened. In this experiment, more anomalies were found using LSTM than RNN except for data in the 1-year training condition, which implies that there is no absolute superiority between RNN and LSTM.

This research also compared the time series algorithms (RNN, LSTM) with the statistical methodology used in the previous research in Section 3.2. First of all, on a weekly training basis, RNN and LSTM reduced anomalies by 44% and 20%, respectively, compared to the statistical approach. Conversely, on a 4-year training basis, RNN and LSTM increased anomalies by 32 and 35 times, respectively. In RNN and LSTM, the 1-week training period showed twice the number of anomalies that the 4-year training period did. However, in the statistical methodology, the number of anomalies is 80 times higher in the 1-week training than in the 4-year training. Using a time series machine learning algorithm effectively controlled the problem of excessive deviation over the training period, which was an issue with previous methodologies. This means that time-series algorithms (RNN and LSTM) can effectively monitor transaction data without being overly influenced by training period.

As a result, this research indicates that training periods and time series machine learning algorithms can be selected depending upon the time and effort available for monitoring. For example, the 4-year training requires more time for learning but the number of anomalies will be small. This allows us to minimize the monitoring range with few anomalies, but also indicates that it might miss some anomalies. A newly launched gambling branch using a 1-week training can still successfully monitor by expending twice the effort used for a 4-year training condition.

### 6.5. Anomaly Detection System for Horse Racing Using Machine Learning

**Figure 9:**
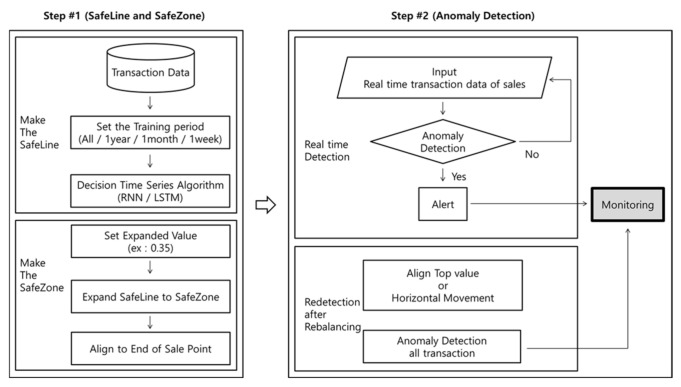
Idea of anomaly detection system for horse racing using machine learning.

Based on these findings, this paper proposes an anomaly detection system (ADS) for horse racing using machine learning algorithms. Figure 9 shows the main idea of the ADS.

The process of detecting anomalies in the ADS is as follows:

- Step #1: As described in Section 5.3.2, a SafeLine is produced using machine learning, and a SafeZone is produced and aligned when sales close. Step #1 can include several types of combinations.
- Step #2: Anomaly detection is processed in real time. Purchased bet data are typed over time. If an anomaly outside the SafeZone is found, the alert system rings, and the anomaly points are monitored. Then, the SafeZone is rebalanced and detections are processed. Similar to the description in Section 5.3.3, the peak values in the transactions of the purchase data and the peak values of the SafeZone overlap or move horizontally. The number of anomalies is then re-estimated, and the detected anomaly points are monitored.

If an anomaly is detected in the monitoring process, an investigation is conducted to detect fraud. During this investigation, the person in charge of monitoring checks the access record of the suspected account, which includes location information. If the account is determined to be fraudulent, the system blocks the account from proceeding further. The ADS ultimately seeks threats that could become a new type of fraud by monitoring the results of anomaly detection. It produces guides for periodic transaction data through machine learning algorithms and can be applied in contexts with accumulated periodic time series transactions.

## 7. Future Work and Conclusions

This paper contributes to anomaly detection in horse racing transactions using IoT based applications. In order to prevent fraudulent activities from occurring, this research investigated ways to reduce the number of flagged anomalies. We examined how the transactions during a race could be improved. Several methodologies for designing an appropriately sized SafeZone were proposed, and the issues regarding the increasing number of anomalies were solved by increasing the training period. This is possible due to time series machine learning algorithms that predict the values of sales in betting tickets. Further studies are needed to investigate the details of weight and ways to decide weight automatically. Another possibility would be to create a flexible SafeZone. This could be achieved in future experiments once more data and cases similar to Walkerhill have been accumulated. While previous research focused on detecting the same types of fradulent activities as demonstrated in the Walkerhill case, this paper has focused on detecting anomalies that will mitigate against a new set of fraudulent cases.

This paper attempted to demonstrate the importance of data analysis for detecting fraudulent activities on IoT devices pertaining to the gambling industry. We hope our experiment and proposed solutions can be applied to real-world problems. Our study found that users may manipulate the IoT-based mobile applications’ data to take advantage of the system. These behaviors further emphasize the need for implementing IoT-based software, data analysis-based solutions, and environments. Moreover, this paper discussed a systematic monitoring solution called the ADS for suggesting practical monitoring solutions for real-world incidents.

## Figures and Tables

**Table 1:** Result of comparison experiments.

	LSTM-AE	SafeZone (LSTM)	SafeZone (RNN)
Accuracy	0.83465	0.91091	0.93705
Precision	0.29514	0.54002	0.62468
Recall	0.42857	0.94883	0.98414
F1 score	0.34956	0.68830	0.76425

**Table 2:** Experimental comparison between RNN, LSTM, and Statistical.

Training	RNN			LSTM			Statistical		
	1st Race	2nd Race	3rd Race	1st Race	2nd Race	3rd Race	1st Race	2nd Race	3rd Race
4 years	57	60	42	72	62	40	2	3	0
1 year	79	97	58	55	57	58	3	3	1
1 month	69	93	82	73	73	127	84	40	3
1 week	83	82	99	88	125	162	236	191	45

## Data Availability

Not applicable.
